# A case of refractory pneumothorax and contralateral atelectasis after thoracoscopic subtotal esophagectomy treated with independent lung ventilation

**DOI:** 10.1186/s40981-022-00537-0

**Published:** 2022-06-29

**Authors:** Natsuko Hirai, Makiko Konda, Yusuke Naito, Nobuhiro Tanaka, Junji Egawa, Masahiko Kawaguchi

**Affiliations:** Department of Anesthesiology, Nara Medical University, 840 Shijo-Cho, Kashihara, Nara, 634-8522 Japan

**Keywords:** Independent lung ventilation, Positive end-expiratory pressures, Pneumothorax, Atelectasis, Pneumonia, Different lung diseases, Case report

## Abstract

**Background:**

Independent lung ventilation (ILV) allows separate positive end-expiratory pressures (PEEP) and inspiratory pressures for each lung. However, only a few articles have reported ILV management for lungs affected by different pathologies.

**Case presentation:**

A 56-year-old man underwent video-assisted thoracic surgery for esophageal cancer. The right lung was injured during surgery, causing a bronchopleural fistula and necessitating chest drainage. On the third day in the intensive care unit, the patient’s oxygenation worsened during pressure support with continuous positive airway pressure ventilation. ILV was initiated for right-sided severe pneumothorax and left-sided atelectasis and pneumonia. ILV was continued for 2 days, and the patient’s trachea was successfully extubated the following day.

**Conclusion:**

Applying high-level PEEP to the one lung and minimizing the airway pressure on the other lung could be achieved using ILV, which might contribute to successful tracheal extubation**.**

## Background

Independent lung ventilation (ILV) is a technique of applying different pressure settings to each lung. Previous research articles [1, 2] have reported the successful use of ILV in the settings of unilateral severe pneumonia or tension pneumothorax. However, there are only a few reports on the use of ILV in patients with bilateral severe conditions and with different diseases on each side [3]. In this case report, we describe a patient with unilateral atelectasis, pneumonia, and a contralateral refractory bronchopleural fistula whose trachea was extubated after successful management with ILV.

## Case presentation

Written informed consent was obtained from the patient prior to the publication of this case report. A 56-year-old male with a history of hypertension and pulmonary emphysema with moderate obstructive respiratory dysfunction underwent video-assisted thoracic surgery for esophageal cancer. Because he had undergone distal gastrectomy with Roux-en-Y anastomosis for gastric cancer 5 years before the current presentation, open residual gastrectomy and ante-thoracic route small bowel reconstruction with vascular anastomosis were scheduled. After the insertion of an epidural catheter, general anesthesia was induced with 80 mg propofol, 100 μg fentanyl, and 50 mg rocuronium, and intubation was performed with a reinforced endotracheal tube (COVIDIEN Shiley ^TM^ 8.5-mm Internal Diameter [I.D.]). Anesthesia was maintained with desflurane, remifentanil, and fentanyl, and neuromuscular blockade was achieved with rocuronium. After anesthesia induction, a bronchial blocker (Phycon TCB blocker type S/T) was placed in the right main bronchus with the aid of fiberscopy. During thoracic manipulation, the balloon of bronchial blocker cuff was inflated with 5 ml of air, and the bronchial blocker was removed after completion of thoracic manipulation.

Surgery was performed under one-lung ventilation. However, the emphysematous lesions of the right lung were torn during surgery, which caused a bronchopleural fistula. It was determined that the air leak was small and that spontaneous closure could be expected by inserting a drainage tube. Moreover, the patient had severe emphysema, and surgical repair would have had more disadvantages. The patient was admitted to the intensive care unit (ICU) with his trachea intubated as continued sedation was necessary to maintain rest at the post-revascularization site.

Air leakage from the right thoracic drain continued upon admission to the intensive care unit. Arterial blood gas analysis immediately on admission showed no acid-base equilibrium abnormalities but poor oxygenation, with an arterial blood oxygen partial pressure (PaO_2_) of 75.1 mmHg, in the setting of assist control (pressure controlled) (A/C [PC]) fraction of inspiratory oxygen (FIO_2_) 0.4, positive end-expiratory pressure (PEEP) 5 cmH_2_O, pressure-controlled ventilation (PCV) 7 cmH_2_O, inspiratory time 1.0 s, and respiratory rate (RR) 8 breaths per minute due to atelectasis caused by sputum accumulation.

**Fig. 1:**
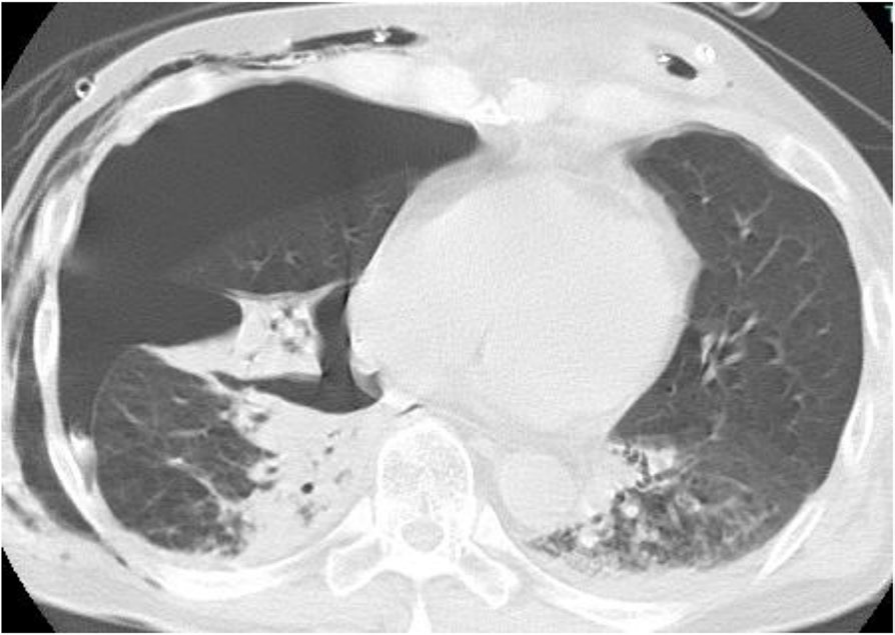
Chest CT scan. Right advanced pneumothorax and left atelectasis and pneumonia visible

**Fig. 2:**
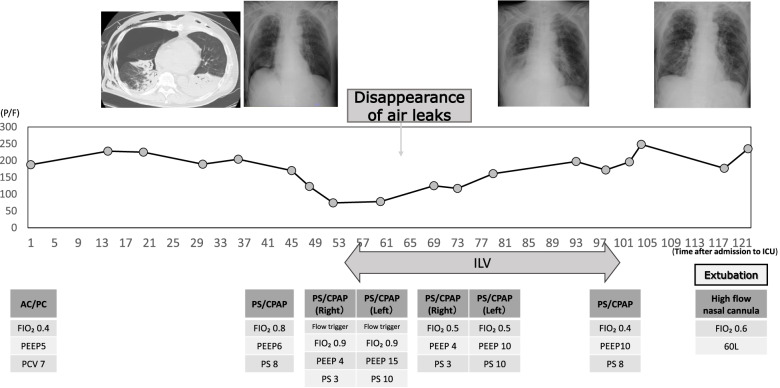
Course of oxygenation from admission, to the intensive care unit, and to extubation. Worsening of oxygenation observed approximately 45 h after admission. After ILV is started, the oxygenation gradually improves and the patient’s trachea is extubated. A/C, assist control; PC, pressure control; PS/CPAP, pressure support with continuous positive airway pressure

Approximately 12 h after admission, the ventilation settings were changed to pressure support with continuous positive airway pressure (PS/CPAP), FIO_2_ 0.4, PEEP 6 cmH_2_O, PS 8 cmH_2_O, and the postoperative P/F ratio (P/F) was approximately 200. The oxygenation gradually worsened from the third day, and a large amount of brownish-purulent sputum was noted predominantly on the left side by bronchoscopy. Oxygenation did not improve, and the P/F ratio decreased to 75. A contrast-enhanced computed tomography (CT) scan of the chest was negative for pulmonary embolization; however, it showed a right-sided severe pneumothorax and dorsal left lower lobe atelectasis and pneumonia (Fig. 1). The patient needed to be managed with increased PEEP to reduce the atelectasis on the left lung and provide respiratory management for pneumonia. However, the air leak during expiration from the right chest drain persisted, contraindicating a PEEP increase. The attending surgeon requested that fistula closure be avoided because of the increased inflammatory response after the previous prolonged surgery. Therefore, the tracheal tube was changed from a single lumen tube (SLT) to a 37-Fr double-lumen tube (DLT) Shiley^TM^ endobronchial tube (Medtronic, Minneapolis, MN, USA), and ILV was initiated using two ventilators (Puritan Bennett™ 840, Medtronic). The tracheal tube exchange was performed under spontaneous breathing without the use of a neuromuscular blocking agent, and ventilation was continued with spontaneous triggering thereafter (Fig. 2). Immediately after the start of ILV, the air leakage during expiration disappeared, and the drainage pressure was changed from −5 to −15 cmH_2_O. Approximately 6 h later, the air leak during inspiration also disappeared. The right lung was expanded, the infiltration shadow was reduced, and the P/F ratio showed improvement. The pressure setting for the left lung was gradually lowered, and ILV was terminated on the fifth day of ICU admission. Since neither pneumothorax nor atelectasis recurred thereafter, the patient’s trachea was extubated and the patient was managed with high-flow nasal cannula oxygen therapy on the sixth day (Fig. 2).

On the 9th day of admission, the patient’s respiratory condition deteriorated due to pneumonia, and his trachea was re-intubated. Extubation was done on the 16th day, and the patient was transferred to the general ward on the 20th day. On the 42nd day, he was discharged home on home oxygen therapy only at night.

## Discussion

ILV has been used in patients with asymmetric lung diseases [4–6]. In the present case, ILV allowed us to maintain adequate ventilation with high PEEP in the left lung while reducing air leakage at the fistula by minimizing airway pressure in the right lung, which led to succesful tracheal extubation. Respiratory management using ILV has been reported in several cases [1–9]. In most cases, ILV was applied for managing unilateral lung disease, such as pulmonary edema following traumatic diaphragmatic hernia surgery or unilateral *Legionella pneumophila* [1, 2, 6, 7]. Only a few reports have described the use of ILV in patients with bilateral lung disease [3].

In our case, in addition to the introduction of ILV, another possible treatment option considered was to close the fistula surgically, initiating respiratory extracorporeal membrane oxygenation (ECMO) or pulmonary physiotherapy to reduce atelectasis while preserving spontaneous breathing, and managing breathing with pressure support ventilation (PSV). However, the attending surgeon requested that fistula closure be avoided because of the increased inflammatory response after the previous prolonged surgery. In addition, adequate oxygenation would be difficult during the one-lung ventilation required for surgery because of atelectasis and pneumonia in the right lung. Initiation of ECMO would also expose the patient to severe additional complications, such as thrombocytopenia, bleeding, and hypotension, even though he met the criteria for ECMO induction according to the guidelines of the Extracorporeal Life Support Organization (ELSO) [10]. Moreover, the patient’s oxygenation had already deteriorated during the approximately 40 h of PSV management, and there was no improvement in oxygenation after sputum suctioning with bronchial fibers suggesting that another treatment should be applied. Consequently, we decided to start ILV; as a result, the air leak stopped shortly afterward, and surgery was avoided.

ILV, however, has several disadvantages. First, the smaller diameter of the DLT compared with that of a single-lumen tube (SLT) increases expiratory resistance, possibly causing expiratory prolongation, even in the absence of obstructive impairment of the preoperative respiratory function. In this case, exhalation resistance, calculated using the inner diameter of the tubes, might have increased 10-fold if we had changed the endotracheal tube from an SLT to a DLT, which could have caused prolongation of the expiratory time. Second, the narrower diameter hinders sputum suctioning compared to when an SLT is used, a disadvantage in the treatment of pneumonia. In this case, a large amount of phlegm had accumulated, but it could be handled by frequent suctioning with a thin suction tube. Finally, it should be noted that the DLT can be easily dislocated by positional changes or other maneuvers. We believe that ILV can be safely performed by keeping it to the shortest possible time.

In this case, the patient underwent tube exchange to a DLT without the use of neuromuscular blocking agents to synchronize left and right ventilation, and he was managed with spontaneous triggering thereafter. When bilateral, synchronized ventilation is provided, patient comfort is maintained, resulting in a reduction in sedative drug administration. In addition, ensuring a phase of low intrathoracic pressure has a positive effect on cardiac function by increasing venous perfusion. In addition, the absence of muscle relaxation can expectantly avoid muscle wasting and atrophy. Conversely, transpulmonary pressure can be controlled by using muscle relaxation; thus, lung-protective ventilation could be achieved. Hence, there could be advantages to its use, especially in patients with ARDS. In our case, since the patient had normal respiratory compliance; it was determinded that muscle relaxants should not be used. In contrast, regarding the issue of synchrony or asynchrony of respiratory phases when performing ILV, previous reports showed that performing ILV asynchronously does not affect oxygenation or circulation [2, 11]; therefore, in the future, asynchronous management may be considered if ILV is used.

In summary, the use of ILV allowed us to achieve the goal of promoting closure of the pulmonary fistula on one side and improving atelectasis on the other, leading to extubation without surgical procedures.

## Data Availability

Data sharing is not applicable to this article as no datasets were generated or analyzed during the current study.

## Abbreviations

I.D.: Internal diameter
ICU: Intensive care unit
PaO_2_: Arterial blood oxygen partial pressure
FiO_2_: Fraction of inspiratory oxygen
A/C (PC): Assist control (pressure controlled)
CT: Computed tomography
DLT: Double-lumen tube
ECMO: Extracorporeal membrane oxygenation
ILV: Independent lung ventilation
OLV: One-lung ventilation
PCV: Pressure-controlled ventilation
PEEP: Expiratory pressure
PS/CPAP: Pressure support with continuous positive airway pressure
PSV: Pressure support ventilation
RR: Respiratory rate
SLT: Single-lumen tube
